# Multifaceted roles of fibronectin type III domain containing 3B (FNDC3B) in cell biology and signaling

**DOI:** 10.3389/fmolb.2026.1741530

**Published:** 2026-02-02

**Authors:** Afreen Khanum, Althaf Mahin, Fathimathul Lubaba, Ashika Bangera, Athira Perunelly Gopalakrishnan, Sowmya Soman, Rajesh Raju

**Affiliations:** Centre for Integrative Omics Data Science (CIODS), Yenepoya (Deemed to be University), Mangalore, Karnataka, India

**Keywords:** adipogenesis, FNDC3B, mutation, phosphosites, transmembrane protein

## Abstract

FNDC3B is an endoplasmic reticulum (ER)-anchored transmembrane protein with diverse roles in cell adhesion, migration, and growth signaling. Recognized as multifunctional, it contributes to key cellular processes such as adhesion, proliferation, differentiation, and migration, yet its molecular functions remain largely unannotated in the Gene Ontology database. Initially identified as a regulator of adipogenesis, promoting fat cell differentiation, FNDC3B also facilitates lung cell maturation, which is essential for neonatal survival. Dysregulation of FNDC3B is implicated in cancer progression through the promotion of epithelial–mesenchymal transition (EMT), metastasis, and modulation of multiple oncogenic signaling pathways. Intriguingly, it exhibits dual roles, acting as either an oncogene or a tumor suppressor, depending on the cellular context. However, the mechanistic determinants of this duality remain elusive. Beyond malignancies, FNDC3B also participates in non-cancerous pathologies, underscoring its broad physiological significance. Although 27 phosphosites have been identified in FNDC3B, the associated signaling networks and functional implications of these modifications remain obscure within the dark phosphoproteome. This review comprehensively delineates the structural, functional, and pathological aspects of FNDC3B, emphasizing its role as a molecular bridge between extracellular and intracellular networks and its growing clinical relevance.

## 1 Introduction

Fibronectin type III domain-containing 3B (FNDC3B), also known as Factor for adipocyte differentiation 104 (FAD104), is an endoplasmic reticulum (ER) transmembrane protein encoded on the long arm (q arm) of chromosome 3, specifically at position 3q26. It has a sequence length of 1,204 amino acids and a molecular weight of 133 kDa (Cai et al., 2012; Fucci et al., 2020). Two major isoforms, arising from alternative splicing, have been reported and differ in sequence length. FNDC3B is one of 11 members of the fibronectin type III domain-containing (FNDC) protein family, characterized by the presence of at least one conserved fibronectin type III (FNIII) domain. FNIII domains are evolutionarily conserved regions of ∼90–100 amino acids, adopting a characteristic β-sandwich fold that contributes to structural stability and protein–protein interactions (Jiang et al., 2022; Ruan et al., 2021). In addition to the FNIII domain, FNDC3B possesses a single transmembrane domain at its C-terminus, which anchors it to the ER membrane and is critical for its function (Nishizuka et al., 2009).

FNDC3B is highly expressed in white adipose tissue, particularly in stromal vascular cells and differentiating adipocytes (Tominaga et al., 2004; Kishimoto et al., 2010), and is moderately expressed in other organ systems, including the endometrium, ovary, and male reproductive tract (Tominaga et al., 2004; Daudon et al., 2022). It was initially recognized for its roles in adipocyte regulation, osteoblast differentiation, and tumor suppression (Kishimoto et al., 2010; Nishizuka et al., 2009). Subsequent studies have implicated FNDC3B in the regulation of cell adhesion, proliferation, migration, signaling, and the epithelial–mesenchymal transition (EMT) (Nishizuka et al., 2009; Han et al., 2020). FNDC3B has been shown to activate several signaling pathways, including integrin, PI3K/Akt, AMPK, TGFβ, and mTOR pathways (Cai et al., 2012). Altered FNDC3B expression has also been reported in several cancers, including pancreatic cancer, cervical cancer, hepatocellular carcinoma, and lung adenocarcinoma (Wang et al., 2024; Han et al., 2020; Lin et al., 2016; Bian et al., 2019). Collectively, these findings highlight FNDC3B as a key component of cellular progression and signaling dynamics.

## 2 FNDC3B structural and functional characteristics

**FIGURE 1:**
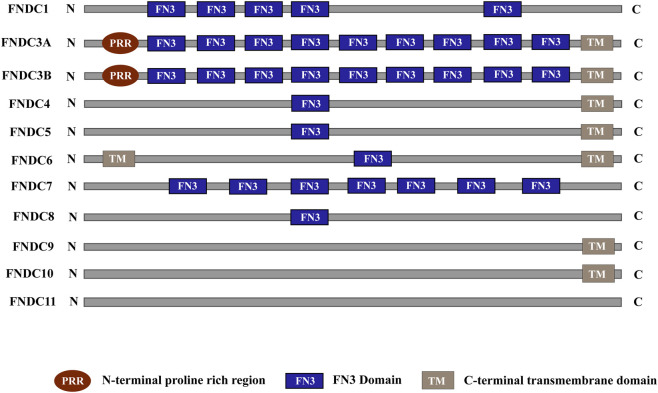
A schematic representation of the Domain architecture of FNDC family members. Domain information was retrieved from the SMART Database.

The fibronectin type III domain-containing (FNDC) protein family consists of 11 members: FNDC1, FNDC3A, FNDC3B, FNDC4, FNDC5, FNDC6 interleukin-20 receptor subunit beta (IL20RB), FNDC7, FNDC8, FNDC9, FNDC10, and FNDC11, each characterized by the presence of at least one conserved fibronectin type III (FNIII) domain, which is illustrated in Figure 1. Although FNDC3B has been implicated in the regulation of adipocyte and osteoblast differentiation, its activation mechanism under physiological conditions remains undefined (Kishimoto et al., 2010). FNDC3B is characterized by nine fibronectin type III (FNIII) domains, a hydrophobic transmembrane segment at its C-terminus, and an N-terminal proline-rich motif (Obholz et al., 2006). Individual FNIII repeats in FNDC3B, approximately 90 amino acids in length, adopt a conserved β-sandwich fold of seven antiparallel β-strands arranged into two sheets. Despite their low sequence identity (∼20–40%), these FNIII domains exhibit high structural homology. FNIII domains constitute one of the largest and most widespread subdomain types in fibronectin, occurring across extracellular matrix proteins, cell-surface receptors, and enzymes (Leahy et al., 1992; Bork and Doolittle, 1992), where they function as structural scaffolds mediating diverse molecular interactions critical for cell adhesion and growth signaling (Lin et al., 2011; Obara et al., 2010). Notably, the first four FNIII domains of FNDC3B have been identified as key mediators of its role in cell migration (Lin et al., 2016).

Considering that, on average any given amino acid makes up about 5% of a protein’s total composition, FNDC3B constitutes ∼13.3% of proline composition in its N-terminal making the N-terminal proline-rich motif (Zhang et al., 2025). This motif imparts structural rigidity through side-chain cyclization with the backbone, thereby stabilizing the three-dimensional conformation and facilitating protein-protein interaction (Adzhubei et al., 2013). At its C-terminus, a hydrophobic transmembrane (TM) domain anchors FNDC3B to the endoplasmic reticulum (ER) membrane, where it participates in essential processes including protein synthesis, folding, trafficking, and ER stress regulation via the unfolded protein response (UPR) (Chi-Rosso et al., 1997; Lin et al., 2016). Both the proline-rich motif and the TM domain are critical for FNDC3B’s metastasis-related function. Notably, deletion of the TM domain shifts its localization from an ER bound cytoplasmic form to the nucleus, resulting in loss of its metastasis-promoting activity (Zhang et al., 2025).

## 3 FNDC3B evolutionary conservation

FNDC3B shares structural similarity with FNDC3A, containing nine fibronectin type III domains, an N-terminal proline-rich motif, and a hydrophobic C-terminal tail. Multiple sequence alignment using the T-Coffee Expresso tool reveals an alignment score of 95, underscoring their close evolutionary relationship. Both proteins contain a conserved N-terminal PPGY motif within a proline-rich region, which serves as a binding site for WW- and SH3-domain-containing proteins (Daudon et al., 2022; Obholz et al., 2006). FNDC3B is also highly conserved across species, including *Homo sapiens*, Neanderthals, *Macaca mulatta*, and *Canis lupus*, with human and mouse FNDC3B sharing 92.5% amino acid sequence similarity (Zhu et al., 2023; Kishimoto et al., 2010). These conserved features highlight the functional importance of FNDC3B in cellular processes relevant to health and disease.

## 4 Regulation of FNDC3B by transcription factors and non-coding RNAs

The expression and activity of FNDC3B are tightly regulated by transcriptional and post-transcriptional mechanisms. Transcription factors such as E2F1 directly bind to the FNDC3B promoter, upregulating its expression and facilitating tumor cell migration and metastasis in hepatocellular carcinoma (HCC). Functional assays demonstrate that E2F1 overexpression significantly enhances FNDC3B levels, while knockdown suppresses its expression and reduces migratory capacity (Hua et al., 2024). In addition, non-coding RNAs, including microRNAs and circular RNAs, serve as important regulators of FNDC3B function. For instance, in cervical cancer, hsa_circ_0001627 promotes tumor progression by sponging miR-1225-5p, a microRNA that normally represses FNDC3B expression. Inhibition of miR-1225-5p by this circRNA elevates FNDC3B expression, which in turn activates PI3K/mTOR signaling to enhance malignancy (Li et al., 2023). Similarly, in colorectal cancer, miR-125a-5p and miR-217 regulate FNDC3B-driven mTOR activation, with increased FNDC3B levels being predictive of poor survival outcomes (Li et al., 2020).

## 5 FNDC3B’s function in cellular dynamics

Dr. Masayoshi Imagawa’s team has extensively studied FNDC3B, where knockdown of its expression was shown to markedly suppress adipogenesis, and FNDC3B-deficient mice died within 1 day of birth, underscoring its essential role in postnatal survival. They further demonstrated that mouse embryonic fibroblasts (MEFs) lacking FNDC3B displayed impaired adipocyte differentiation, reduced proliferation, and compromised cytoskeletal organization, leading to defective stress fibre formation and delayed adhesion, spreading, and migration (Nishizuka et al., 2009). Importantly, FNDC3B was found to reciprocally regulate mesenchymal differentiation, functioning as a positive regulator of adipogenesis and a negative regulator of osteogenesis (Kishimoto et al., 2010). Their phenotypic and morphological analyses revealed that neonatal lethality resulted from cyanosis-associated lung dysplasia, including atelectasis, and immunohistochemical studies identified strong FNDC3B expression in alveolar type II (ATII) cells, confirming its indispensable role in lung maturation and ATII cell differentiation (Kishimoto et al., 2011). In the context of osteogenesis, they showed that FNDC3B disruption caused craniosynostosis-like premature calvarial ossification, and primary calvarial cell analyses established FNDC3B as a negative regulator of the BMP/Smad signaling pathway. Mechanistically, the N-terminal proline-rich motif of FNDC3B directly interacted with Smad1/5/8, thereby attenuating their phosphorylation and downstream transcriptional activity (Chen et al., 2012; Kishimoto et al., 2013).

Extending their research beyond development, Dr. Imagawa’s team demonstrated that FNDC3B is also implicated in tumor progression. In human cervical cancer HeLa cells, FNDC3B expression was upregulated during TGF-β–induced epithelial-to-mesenchymal transition (EMT). Knockdown of FNDC3B enhanced TGF-β–mediated EMT and migration, whereas overexpression suppressed EMT. Mechanistic studies revealed that FNDC3B negatively regulated Smad2/3 phosphorylation but positively regulated Smad1/5/8 phosphorylation during EMT (Goto et al., 2017). In melanoma, FNDC3B was shown to suppress invasion and metastasis by inhibiting STAT3 signaling. Its expression was lower in highly metastatic A375SM cells than in poorly metastatic A375C6 cells, and reduction of FNDC3B enhanced, while overexpression suppressed melanoma cell migration and invasion. Stable expression of FNDC3B further reduced lung colonization *in vivo*, consistent with its role as a negative regulator of STAT3 phosphorylation and transcriptional activity, without affecting total STAT3 protein levels (Katoh et al., 2015). Mechanistically, FNDC3B was found to interact with the C-terminal region of STAT3, and deletion analysis confirmed that its N-terminal region is indispensable for STAT3 inhibition and suppression of anchorage-independent melanoma growth, thereby establishing FNDC3B as a critical negative regulator of malignant transformation (Katoh et al., 2016).

FNDC3B has also been detected in stress granules that assemble under conditions of cellular stress (Jain et al., 2016). These dense ribonucleoprotein aggregates, composed of mRNAs and proteins, serve as transient sites for translational regulation. Consistent with this, FNDC3B has been identified as a candidate RNA-binding protein (Castello et al., 2012).

## 6 Cellular signaling associated with FNDC3B

A comprehensive understanding of cellular signaling is fundamental to deciphering protein function, as these networks govern the spatial and temporal regulation of activity, interactions, and downstream outcomes in both physiological and pathological contexts. Among these mechanisms, phosphorylation serves as a dynamic switch that modulates protein activity, interactions, and localization, thereby orchestrating cellular responses to environmental cues. Mapping phosphosignaling patterns is essential to identify functional signaling networks, understand their perturbations in disease, and develop targeted therapeutic strategies (Krug et al., 2019). According to the PhosphoSitePlus database, 27 phosphosites have been identified in human FNDC3B, with the majority located in the N-terminal and C-terminal regions (Hornbeck et al., 2019). The molecular significance of none of these phosphosites is currently known, and the kinases mediating these phosphorylations have also not been identified, rendering FNDC3B an enigmatic protein within the dark phosphoproteome.

Further, based on our research interest, we examined the phosphosites of FNDC3B that were differentially expressed across global cellular phosphoproteomic datasets previously curated and processed in our laboratory (Mahin et al., 2025; Khan et al., 2025). This heterogeneous pool of data was obtained from PubMed-indexed studies conducted under diverse experimental conditions and includes multitemporal datasets generated by researchers worldwide. Across this dataset, we identified 7 phosphosites of FNDC3B that were differentially expressed, with a minimum standard cutoff of a p-value <0.05 and a fold change of ≥1.3 for upregulation and ≤0.76 for downregulation. The phosphosites identified in this dataset exhibited a pattern of occurrence similar to that reported in the PhosphoSitePlus database, with six of the seven sites located in the N-terminal region of FNDC3B. The frequency of their differential expression varied, with phosphosites S211, S260, S257, S254, S238, S393, and S208 identified as differentially expressed in 3, 3, 6, 2, 4, 5, and 74 datasets, respectively (Supplementary Table S1). Interestingly, phosphorylation at S208 of FNDC3B appears to play a predominant role in its signaling compared to other phosphosites, as it is reported to be detected across 45 high-throughput datasets in the PhosphoSitePlus database and was identified to be differentially expressed in 74 cellular phosphoproteomic datasets in our analysis. Differential expression of S208 was observed under diverse experimental conditions, including cancer-associated cell line studies such as lung adenocarcinoma, glioblastoma, breast cancer, squamous cell carcinoma, and colorectal cancer. However, the molecular significance of phosphorylation at S208 and the kinase responsible for this modification remain poorly characterized.

**FIGURE 2:**
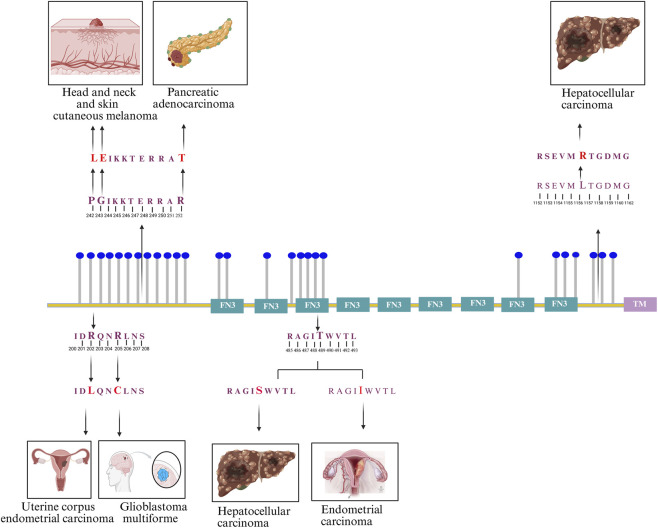
Schematic representation of 27 FNDC3B phosphosites reported in the PhosphoSitePlus database, with depictions of mutations associated with distinct cancer types occurring within phosphomotifs. These include R202L (uterine corpus endometrial carcinoma), R205C (glioblastoma multiforme), G243E and P242L (head and neck and skin cutaneous melanomas), R252T (pancreatic adenocarcinoma), T489S and T489I (hepatocellular and endometrial carcinomas), and L1157R (hepatocellular carcinoma).

Furthermore, analysis of FNDC3B-associated data from the ActiveDriver database, which integrates genetic variation in human and cancer genomes with post-translational modification sites and signaling networks, revealed 13 mutations located adjacent to phosphosites or within the phosphomotifs of nine FNDC3B phosphosites, which have been implicated in various cancers (Krassowski et al., 2021). These mutations include R202L in uterine corpus endometrial carcinoma, R205C in glioblastoma multiforme, G243E and P242L in head and neck and skin cutaneous melanomas, R252T in pancreatic adenocarcinoma, T489S and T489I in hepatocellular carcinoma and endometrial carcinoma, and L1157R in hepatocellular carcinoma. The schematic overview of phosphosites and the mutation adjacent to phosphosites or within the phosphomotifs, which are associated with various cancers, is represented in Figure 2. These reported variations associated with FNDC3B phosphosites and their clinical relevance warrant detailed analysis of FNDC3B-associated cellular phosphosignaling patterns.

## 7 Pathological significance associated with FNDC3B

FNDC3B has emerged as a context-dependent regulator of cancer biology, displaying both oncogenic and tumor-suppressive properties. Aberrant overexpression of FNDC3B has been documented in multiple malignancies, including hepatocellular carcinoma (HCC), acute myeloid leukemia (AML), tongue squamous cell carcinoma, colorectal, ovarian, breast, cervical, and pancreatic cancers (Han et al., 2020; Chen et al., 2010; Wang X. et al., 2022). Elevated FNDC3B expression is consistently linked to poor prognosis, including reduced progression-free intervals and overall survival. In pancreatic cancer, it is notably proposed as a prognostic biomarker and therapeutic target due to its markedly higher expression in tumor tissues compared with adjacent normal tissues (Wang et al., 2024). In hepatocellular carcinoma, FNDC3B promotes oncogenic signaling by interacting with annexin A2 (ANXA2) to remodel actin cytoskeletal architecture, thereby driving cell motility and metastatic potential (Lin et al., 2016). In gastric cancer, FNDC3B directly interacts with and stabilizes Family with Sequence Similarity 83 Member H (FAM83H), preventing its proteasomal degradation and consequently activating the Snail-driven EMT program to promote invasion and metastasis (Zhang et al., 2025). Conversely, in melanoma, FNDC3B has been reported to exert tumor-suppressive activity by inhibiting STAT3 phosphorylation and downstream transcriptional activation (Kwon et al., 2023).

Beyond cancer, FNDC3B also participates in non-malignant diseases, further underscoring its pleiotropic biological functions. Genome-wide association studies (GWAS) have linked FNDC3B to keratoconus, with the SNP rs4894535 showing a significant association (OR = 1.47, 95% CI = 1.29–1.68, P = 4.9 × 10^−9^), highlighting its contribution to disease susceptibility (Lu et al., 2013; Sahebjada et al., 2013; Rong et al., 2017). GWAS have also implicated FNDC3B in intraocular pressure regulation and primary open-angle glaucoma, with significant association at chromosome 3q25.31 (rs6445055, P = 4.19 × 10^−8^), alongside additional loci in ABCA1, ABO, and chromosome 11p11.2 (Hysi et al., 2014; Wiggs and Pasquale, 2017). An association of SNP in FNDC3B (rs7636836) with pseudoexfoliation glaucoma among men, and an increased risk of primary open-angle glaucoma have been reported in the Japanese and Middle-Eastern Saudi cohorts (Shiga et al., 2018; Kondkar et al., 2022). FNDC3B is also linked to sporadic Parkinson’s disease, with GWAS showing strong association (Hu et al., 2016). A rare chromosomal translocation t(3; 17) (q26; q21) gives rise to the FNDC3B-RARA fusion gene in a variant form of acute promyelocytic leukemia (APL). This fusion links exon 24 of FNDC3B with exon 3 of retinoic acid receptor alpha (RARA), producing chimeric transcripts that repress retinoic acid-responsive elements, impair granulocytic differentiation, and promote leukemogenesis (Cheng et al., 2017; Mannan et al., 2020).

**FIGURE 3:**
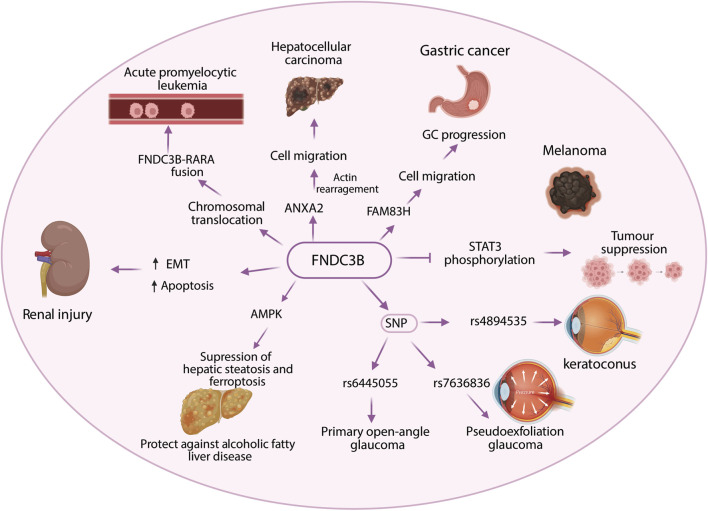
Diagram illustrating FNDC3B interactions affecting various conditions. Arrows show pathways connecting FNDC3B to acute promyelocytic leukemia, renal injury, hepatocellular carcinoma, gastric cancer, melanoma, glaucoma types, and keratoconus. Elements include chromosomal translocation, cell migration, STAT3 phosphorylation, and genetic variants, emphasizing FNDC3B’s role in diverse diseases.

FNDC3B is also associated with metabolic and organ specific disorders. Kei Tominaga et al. demonstrated FNDC3B as a positive regulator of adipogenesis. He observed a transient expression of FNDC3B at the earlier stages of adipocyte differentiation. Further, knockdown of FNDC3B by RNAi in 3T3-L1 cells significantly declined its ability to differentiate to mature adipocytes (Tominaga et al., 2004). Moreover, regulation of FNDC3B by miR-215-5p has been reported to inhibit adipocyte differentiation of 3T3-L1 cells (Peng et al., 2016). Although its role in adipocyte differentiation can be considered as a plausible attributing factor to obesity, a thorough characterization of the functional role of FNDC3B in obesity is still missing. However, with the positive energy balance considered as a primary cause of obesity, GWAS of individuals of European ancestry from the Atherosclerosis Risk in Communities and UK Biobank populations analyzed by Gulisija et al, genomic variants in *FNDC3B* loci were found associated with distinct sets of energy balance-contributing traits such as physical activity and increased hip circumference (rs582780) (Gulisija et al., 2025). Furthermore, FNDC3B has been reported to alleviate hepatic steatosis and ferroptosis via activation of AMP-activated protein kinase (AMPK) (You et al., 2022), whereas in renal ischemia–reperfusion injury, it promotes tubular epithelial apoptosis through TGF-β1 signaling, an effect antagonized by the protective lncRNA XLOC_032768 (Zhou et al., 2020). Despite accumulating reports on the involvement of FNDC3B in various cancers, its role in metabolic diseases and its potential as therapeutic target remains largely unexplored and warrants further investigations. The involvement of FNDC3B in diverse pathological conditions is represented in Figure 3.

## 8 Circular FNDC3B in oncology and beyond

FNDC3B is also identified in the form of circular RNA. circFNDC3B is derived from exons 5 and 6 of the *FNDC3B* gene that exhibits high cytoplasmic stability with a half-life exceeding 24 h (Zeng et al., 2020). Functionally, it regulates gene expression through diverse mechanisms, including sponging oncogenic or tumour-suppressive miRNAs (Tang et al., 2022) and encoding a 218-amino-acid peptide (circFNDC3B-218aa) that modulates cancer progression and metabolic pathways (Pan et al., 2020). Its role in tumor biology is highly context-dependent. In bladder cancer, circFNDC3B is downregulated and acts as a tumor suppressor by modulating the miR-1178-3p/G3BP2 axis (Liu et al., 2018). Similarly, in colorectal cancer, reduced expression of circFNDC3B exerts inhibitory effects on epithelial–mesenchymal transition (EMT), angiogenesis, and metastasis via the miR-937-5p/TIMP3 pathway, in addition to producing circFNDC3B-218aa with tumor-suppressive activity (Zeng et al., 2020; Zeng et al., 2022). In contrast, circFNDC3B is markedly upregulated in several malignancies, where it assumes oncogenic functions. In gastric cancer, it facilitates migration, invasion, and EMT through the IGF2BP3/CD44 signaling axis (Hong et al., 2019; Zhang et al., 2022). In esophageal squamous cell carcinoma, circFNDC3B promotes proliferation and migration by regulating the miR-490-5p/TXNRD1 and miR-214-3p/CDC25A pathways (Tang et al., 2022; Wang J. et al., 2022). In renal carcinoma, it contributes to curcumin-mediated tumor suppression by modulating the miR-138-5p/IGF2 axis (Xue et al., 2021), while in oral squamous cell carcinoma it enhances malignant phenotypes by suppressing ferroptosis through ceRNA networks such as miR-520d-5p/SLC7A11 and miR-1322/MED1 (Yang et al., 2021).

Emerging evidence also implicates circFNDC3B in non-malignant diseases. In cardiovascular disease, circFNDC3B is significantly downregulated following myocardial infarction, where it promotes cardiac repair by enhancing VEGF-A expression through interaction with the RNA-binding protein FUS (Fused in Sarcoma) (RBP FUS) (Garikipati et al., 2019). Conversely, in abdominal aortic aneurysm, circFNDC3B expression is elevated and contributes to vascular pathology by regulating inflammatory and oxidative stress responses via the miR-143-3p/ADAM10 axis (Liu et al., 2021). Taken together, circFNDC3B acts as a context dependent multifunctional regulator with dual oncogenic and tumor-suppressive properties in cancer, while also exerting critical effects in non-cancerous conditions (Sun et al., 2023).

## 9 Preclinical and clinical aspects of FNDC3B

To the best of our knowledge, there are no active or completed clinical trials directly targeting FNDC3B. Nevertheless, FNDC3B has become the subject of extensive preclinical investigation, primarily in cell-based systems and animal models, and is increasingly recognized as a potential therapeutic target and prognostic biomarker in multiple malignancies. Genetic knockout studies in mice reveal that loss of FNDC3B results in perinatal lethality accompanied by defects in adipocyte differentiation, underscoring its essential role in development and tissue homeostasis (Kishimoto et al., 2011). A substantial body of preclinical evidence supports a context-dependent oncogenic role for FNDC3B across diverse disease models. Elevated FNDC3B expression has been consistently reported in hepatocellular carcinoma, cervical cancer, gastric cancer, lung adenocarcinoma, and glioma, where it correlates with increased cellular proliferation, migration, invasion, acquisition of EMT like phenotypes, stemness-associated traits, and unfavorable clinical outcomes. A preliminary analysis demonstrated the prognostic as well as therapeutic potential of FNDC3B in pancreatic cancer (Wang et al., 2024) Integrated machine learning approaches revealed that FNDC3B can be used as a prognostic and immunotherapeutic biomarker in glioma (Wang X. et al., 2022). Kwon et al. illustrates that FNDC3B could be a promising therapeutic target in GBM patients (Kwon et al., 2023). FNDC3B was found to interact with FAM83H (a regulatory protein involved in EMT) and prevents the ubiquitin-proteasome degradation of FAM83H which in turn promotes gastric cancer progression (Zhang et al., 2025). The expression of FNDC3B is found enhanced in cancers including cervical cancer as well as hepatocellular carcinoma and leads to its metastasis (Han et al., 2020; Lin et al., 2016). Though several reports highlight the potential of FNDC3B as a biomarker and therapeutic target in diverse cancers, the exact function and mechanism of FNDC3B in cancer progression is still unclear. An integrated bioinformatic study by Wu et al highlights FNDC3B as a key regulatory gene involved ECM-receptor interaction, ECM remodelling and immune infiltration in oral squamous cell carcinoma and periodontal disease (Wu et al., 2025). A comprehensive analysis identified E2F1 as a transcriptional activator of FNDC3B and is involved in cell migration in hepatocellular carcinoma (Hua et al., 2024). Functional studies further demonstrate that genetic silencing or knockdown of FNDC3B significantly suppresses tumor growth, invasive capacity, and metastatic dissemination in both *in vitro* assays and xenograft or orthotopic animal models, reinforcing its functional relevance in tumor progression (Lin et al., 2016; Bian et al., 2019; Wang J. et al., 2022; Zhang et al., 2025). Fusion of FNDC3B with other genes such as PRKCI, EVI1 and RARA is implicated in multiple cancers and reinstates the significance of FNDC3B in pathophysiological processes (Hua et al., 2017; Wang et al., 2016; Cheng et al., 2017). Analyses of large-scale transcriptomic datasets derived from clinical samples, including those from The Cancer Genome Atlas and the Human Protein Atlas, consistently associate elevated FNDC3B expression with reduced overall and disease-free survival across multiple cancer types. High FNDC3B expression further correlates with adverse clinicopathological features, such as higher tumor grade, advanced disease stage, and lymph node metastasis (Wang X. et al., 2022; Jiang et al., 2022; Kwon et al., 2023).

## 10 Current strategies for targeting FNDC3B

While FNDC3B represents a potentially attractive therapeutic target, no specific or clinically approved small-molecule inhibitors of FNDC3B are currently available. Moreover, the absence of an experimentally resolved crystal structure for FNDC3B has constrained structure-based drug discovery efforts for candidate small-molecule inhibitors. Consequently, the identification of FNDC3B-directed lead compounds remains a significant unmet challenge. Current preclinical studies have predominantly employed genetic and transcript-level strategies involving siRNA, shRNA, antisense oligonucleotides (ASOs), and CRISPR/Cas9 to study FNDC3B-driven cellular phenotypes (Fan et al., 2013; Liedtke et al., 2019). At the protein level, structural and functional analyses reveal that FNDC3B harbours fibronectin type III domains and a proline-rich N-terminal region that mediate interactions with cytoskeletal and membrane-associated proteins, notably annexin A2 (ANXA2), which are critical for cell motility and invasive behaviour (Lin et al., 2016). Accordingly, disruption of these protein–protein interactions using peptide-based inhibitors, small-molecule PPI inhibitors, or engineered biologics represents a plausible therapeutic strategy.

In parallel, FNDC3B has been linked to activation of the PI3K/AKT/mTOR signaling axis, regulation of epithelial–mesenchymal transition, cytoskeletal remodelling, and endoplasmic reticulum stress responses (Li et al., 2020), indicating that indirect pathway-based interventions may also mitigate FNDC3B-driven phenotypes. In this context, pharmacological inhibitors targeting PI3K, AKT, mTOR, focal adhesion kinase (FAK), or Rho/ROCK signaling pathways offer immediately actionable avenues for therapeutic modulation. Furthermore, emerging proteolysis-targeting strategies, including proteolysis-targeting chimeras (PROTACs) and molecular glue degraders, provide a conceptual framework for selectively eliminating FNDC3B at the protein level, contingent upon the identification of suitable binding ligands, and may offer advantages over functional inhibition alone (Li et al., 2020; Zhao et al., 2022). A summary table depicting the functional role, molecular signaling, pathological relevance and current strategies for targeting FNDC3B is represented in Table 1.

**TABLE 1:** Summary of functional roles, molecular signaling pathways, pathological relevance, and current strategies for targeting FNDC3B is provided in the table.

Categories of discussion	Biological context	Mechanistic insight	References
Functional roles	Adipogenesis	Positive regulator of adipocyte differentiation	Kishimoto et al. (2010)
	Osteogenesis	Negative regulator of osteoblast differentiation	Kishimoto et al. (2010)
	Cell adhesion and migration	Controls cytoskeletal organization, stress fiber formation, and cell spreading	Lin et al. (2011), Obara et al. (2010), Nishizuka et al. (2009)
	Epithelial–mesenchymal transition (EMT)	Context-dependent EMT regulation via TGF-β and Snail-associated pathways	Goto et al. (2017)
Molecular pathways	TGF-β/Smad signaling	FNDC3B negatively regulated Smad2/3 phosphorylation but positively regulated Smad1/5/8 phosphorylation during EMT	Goto et al. (2017)
	PI3K/Akt/mTOR	Promotes tumor progression in multiple cancers	Cai et al. (2012), Li et al. (2023), Li et al. (2020)
	AMPK	Protective role in hepatic steatosis and ferroptosis	You et al. (2022)
Pathologic Conditions	Oncogenic roles	HCC, pancreatic, gastric, colorectal, cervical, ovarian, breast cancers (EMT activation, cytoskeletal remodeling, mTOR signaling)	Lin et al. (2016), Wang et al. (2024), Zhang et al. (2025), Han et al. (2020), Chen et al. (2010), X. Wang J. et al. (2022)
	Acute Promyelocytic Leukemia	FNDC3B–RARA fusion	Cheng et al. (2017), Mannan et al. (2020)
	Tumor-suppressive roles	Melanoma (Inhibits STAT3 phosphorylation and metastasis)	Kwon et al. (2023)
	Non-cancer Diseases a. Alcoholic fatty liver disease	Alleviates hepatic steatosis via AMPK	You et al. (2022)
	b. Renal injury	Promotes apoptosis via TGF-β1 signaling	Zhou et al. (2020)
	c. Eye diseases	Keratoconus, glaucoma (GWAS)	Lu et al. (2013), Sahebjada et al. (2013), Rong et al. (2017), Hysi et al. (2014), Wiggs and Pasquale (2017)
Circular RNA (circFNDC3B)	Oncogenic roles	Gastric, ESCC, oral cancer	Hong et al. (2019), Zhang et al. (2022), Tang et al. (2022), J. Wang X. et al. (2022), Yang et al. (2021)
	Tumor suppressor roles	Bladder, colorectal cancer	Liu et al. (2018), Zeng et al. (2020), Zeng et al. (2022)
	Non-cancer roles	Cardiac repair, vascular inflammation	Garikipati et al. (2019), Liu et al. (2021)
Cellular Signaling	Known phosphosites	27 sites reported in PhosphoSitePlus	Hornbeck et al. (2019)
	Predominant phosphosite	S208 is most frequently detected across datasets	Mahin et al. (2025)
	Cancer-associated mutations	13 mutations located adjacent to phosphosites or within the phosphomotifs	Krassowski et al. (2021)
Current strategies for targeting FNDC3B	Genetic and transcript-level strategies	siRNA, shRNA, antisense oligonucleotides (ASOs), and CRISPR/Cas9	Fan et al. (2013), Liedtke et al. (2019)
	Protein–protein interaction targeting	Peptide-based inhibitors, small-molecule PPI inhibitors, or engineered biologics	Lin et al. (2016)
	Pathway-based therapeutic approaches	PI3K, AKT, mTOR, FAK, Rho/ROCK inhibitors	Li et al. (2020)
	Proteolysis-targeting strategies	Proteolysis-targeting chimeras (PROTACs) and molecular glue degraders	Li et al. (2020), Zhao et al. (2022)

## 11 Future directions and conclusion

FNDC3B has emerged as a multifunctional protein with critical roles in cellular processes and diverse pathological conditions, particularly in cancer. However, its precise molecular functions remain insufficiently understood. Defining the mechanisms that govern FNDC3B localization, interactions, and signaling is a key priority. In particular, the transmembrane domain warrants focused investigation, as it anchors FNDC3B to the endoplasmic reticulum, a localization essential for promoting cell migration and epithelial–mesenchymal transition (EMT). Loss of this domain causes nuclear mislocalization and abrogates its migration-inducing activity, underscoring its functional importance. Equally important is mapping the kinase signaling pathways that regulate FNDC3B through phosphorylation, as the kinases responsible for modifications at key sites, such as S208, remain unidentified.

FNDC3B is increasingly recognized as an oncogene driving cancer progression and metastasis across multiple tumor types. Future work should prioritize elucidating its signaling networks, post-translational modifications, and protein–protein interactions, which are crucial for the development of targeted therapies. Additionally, functional characterization of FNDC3B-derived circular RNAs and their encoded peptides will provide insights into their context-dependent tumor-suppressive or oncogenic roles. FNDC3B’s emerging involvement in immune modulation also highlights its potential as an immunotherapeutic target, particularly in reshaping the tumor microenvironment. Finally, a detailed analysis of the phospho-signaling patterns that alter FNDC3B at the molecular level, as well as the kinases that mediate these phosphorylations, needs to be explored to better comprehend FNDC3B for therapeutic and clinical interventions.
